# Revisiting immune escape in colorectal cancer in the era of immunotherapy

**DOI:** 10.1038/s41416-019-0421-x

**Published:** 2019-03-13

**Authors:** Marieke Erica Ijsselsteijn, Florent Petitprez, Laetitia Lacroix, Dina Ruano, Ruud van der Breggen, Catherine Julie, Hans Morreau, Catherine Sautès-Fridman, Wolf Herman Fridman, Noel Filipe da Cunha Carvalho de Miranda

**Affiliations:** 1 Department of Pathology, Leiden University Medical Center, 2333ZA Leiden, The Netherlands; 2 INSERM, UMR_S 1138, Cordeliers Research Center, Team Cancer, Immune Control and Escape, 75006 Paris, France; 3 University Paris Descartes Paris 5, Sorbonne Paris Cite, UMR_S 1138, Centre de Recherche des Cordeliers, 75006 Paris, France; 4 University UPMC Paris 6, UMR_S 1138, Sorbonne University, 75006 Paris, France; 5 Programme Cartes d’Identité des Tumeurs, Ligue Nationale Contre le Cancer, Paris, France; 6 Laboratoire d’anatomie pathologique, Hopital Ambroise Paré, AP-HP, Boulogne, France

**Keywords:** Immune evasion, Oncogenesis, Immunoediting, Colorectal cancer, Metastasis

## Abstract

In colorectal cancer (CRC), T-cell checkpoint blockade is only effective in patients diagnosed with mismatch repair-deficient (MMR-d) cancers. However, defects in Human Leukocyte Antigen (HLA) class I expression were reported to occur in most MMR-d CRCs, which would preclude antigen presentation in these tumours, considered essential for the clinical activity of this immunotherapeutic modality. We revisited this paradox by characterising HLA class I expression in two independent cohorts of CRC. We determined that loss of HLA class I expression occurred in the majority (73–78%) of MMR-d cases. This phenotype was rare in CRC liver metastases, irrespective of MMR status, whereas weak, inducible expression of HLA class I molecules was frequent in liver lesions. We propose that HLA class I is an important determinant of metastatic homing in CRCs. This observation is paramount to understand CRC carcinogenesis and for the application of immunotherapies in the metastatic setting.

## Introduction

T-cell checkpoint blockade immunotherapies have recently emerged as revolutionary cancer treatments.^1^ Antibodies targeting the CTLA-4 receptor and the PD-1/PD-L1 axis reinvigorate T-cell-mediated cancer immunity, particularly in cancers with high mutation burden.^2^ In such malignancies, there is a high likelihood that neo-antigens are presented in complex with Human Leukocyte Antigen class I (HLA class I) at the surface of tumour cells and by antigen presenting cells. Not surprisingly, in colorectal cancer (CRC), checkpoint blockade therapies are thus far only effective in a proportion of mismatch repair-deficient (MMR-d) CRCs.^3^

Conspicuous infiltration by cytotoxic T-cells is a hallmark of MMR-d CRCs as a consequence of their immunogenic character.^4^ Hence, these tumours are also prone to acquire phenotypes that result from immunoediting processes; loss of HLA class I expression was described to occur in up to 60% of MMR-d CRCs.^5,6^ This observation is somewhat paradoxical with the responses observed to checkpoint blockade in MMR-d CRC patients, assuming that neo-antigen presentation by tumour cells is required for therapy-induced T-cell responses. We have revisited this paradox by characterising, in detail, HLA class I phenotypes in two independent CRC cohorts.

## Methods

### Patient samples

HLA class I expression was assessed in a Dutch (NL, *N* = 208) and a French (FR, *N* = 45) cohort of archival CRC tissues. In the FR cohort, MMR protein expression (MLH1, MSH2, MSH6, and PMS2) was analysed by immunohistochemistry. MMR protein loss was defined as the absence of nuclear staining in neoplastic cells but positive nuclear staining in lymphocytes and normal adjacent colonic epithelium. Tumours deemed MMR-d underwent microsatellite instability (MSI) analysis using five monomorphic mononucleotide markers (BAT-25, BAT-26, NR-21, NR-24, and NR-27). Specimens with at least three unstable markers were scored as highly unstable and therefore MMR-d. In the NL cohort, the MMR status was determined by means of the MSI Analysis System Version 1.2 (Promega, WI, USA) according to the manufacturer’s instructions. DNA analysis could not be performed in 20% of cases in the cohort that were instead interrogated by anti-PMS2 and anti-MSH6 immunohistochemistry, followed by MLH1 and MSH2 detection in case of aberrant immunodetection. RNA expression data and resulting consensus molecular subtypes (CMS) classification was available for the majority (*N* = 40) of cases in the FR cohort.

### Immunohistochemistry

**Fig. 1:**
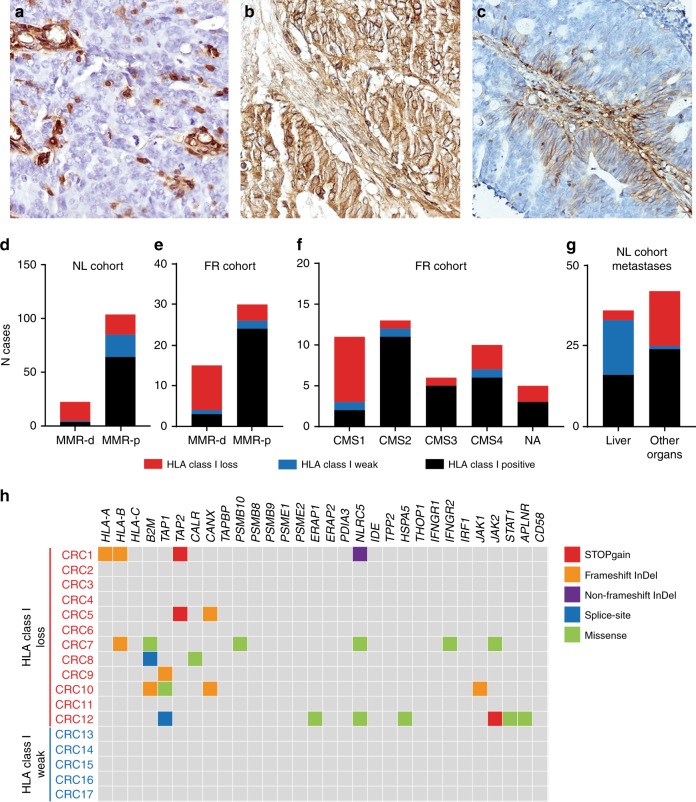
HLA class I phenotypes were determined by immunohistochemistry and categorised into lost (**a**), regular (**b**), and weak (**c**) membranous HLA class I expression. The distinct HLA class I phenotypes were differentially distributed amongst MMR-d and MMR-p CRCs in both NL (**d**) and FR (**e**) cohorts, CMS subtypes (**f**), and metastatic sites (**g**). Genetic alterations in HLA class I genes and other genes involved in antigen presentation were determined in tumours with absent and weak HLA class I expression (**h**)

Immunodetection of HLA class I expression was performed with the HCA2 and HC10 monoclonal antibodies (Nordic-MUbio, The Netherlands) as reported previously,^5^ with some modifications. The procedure included heat-mediated antigen retrieval in 10 mM citrate buffer (pH 6.0) for 10 min and endogenous peroxidase blocking in a 0.3% hydrogen peroxidase/methanol solution. The HCA2 and HC10 antibodies were applied overnight at a concentration of 0.3 μg/mL in PBS with 1% BSA. Detection of primary antibody binding was performed with a polymeric HRP-linker antibody conjugate (Immunologic, The Netherlands) and DAB+chromogen (Dako, Agilent Technologies, CA, USA). HLA class I loss was defined by the lack of HLA class I expression (determined by either of the antibodies) at the membrane of tumour cells, accompanied by retained expression in tumour-infiltrating immune cells (Fig. 1a). In addition to regular HLA class I expression (Fig. 1B), a third phenotype, defined as weak HLA class I expression, corresponded to limited membranous expression of HLA class I that was only upregulated in the presence of infiltrating immune cells (Fig. 1c). The scoring of HLA class I phenotypes was performed blindly in relation to the MMR status.

### Exome sequencing

Detailed procedures on exome sequencing are presented as supplementary information.

## Results

We observed loss of membranous HLA class I expression in 29 and 33% of CRC cases from the NL and FR cohorts, respectively. This phenotype was most prevalent in the immunogenic MMR-d subsets, occurring in 78% (Fig. 1d, NL cohort) and 73% (Fig. 1e, FR cohort) of MMR-d tumours, respectively, as well as in the CMS1 subtype (in 73%, Fig. 1f). While the CMS1 CRC subtype is known to be mainly composed out of MMR-d tumours, it has been described as a group presenting elevated levels of HLA class I expression, based on RNA expression data.^7^ These data can be conciliated by the following observations: (1) MMR-d and CMS1 tumours are conspicuously infiltrated by immune and other stromal cells expressing high levels of HLA class I that are detected by RNA expression analysis (Fig. 1a); (2) the exposure to an inflammatory environment might be responsible for upregulating HLA class I gene expression in tumour cells but the presence of HLA class I molecules at the cell surface is precluded by defects in other components of the antigen processing machinery (Fig. 1c).^5,6^

Strikingly, the distinct HLA class I phenotypes (lost, regular, and weak HLA class I expression, Fig. 1a–c) were differentially distributed according to metastatic sites in stage IV CRCs. Weak HLA class I expression was observed in 49% of liver metastasis while rarely observed at other metastatic sites (Fig. 1g). Conversely, cancers that lost HLA class I expression rarely metastasized to the liver and were most often found at other organs (Fig. 1g). Of note, MMR-d metastatic lesions were rare (*N* = 4) and therefore these observations mainly rely on MMR-p tumours. The weak HLA class I expression phenotype described here has been previously considered to constitute a defect in antigen presentation as judged from reports indicating a high incidence of HLA class I defects in CRC liver metastases.^8^ We performed exome sequencing in 12 CRC samples that lost HLA class I expression and five tumours which presented weak HLA class I expression, for which frozen tissue was available, in order to clarify the genetic basis of these phenotypes. We interrogated the presence of mutations in the HLA class I genes, *B2M*, and other genes known to be involved in antigen processing and regulation of HLA class I expression (Fig. 1h). We did not observe mutations in any of the 30 investigated genes in the samples with weak HLA class I expression, thus indicating that this phenotype does not correspond to an HLA class I defect caused by genetic aberrations. On the other hand, deleterious mutations were found in 7 (58%) tumours that lost HLA class I expression (Fig. 1h). Given the prevalence of HLA class I defects in MMR-d and the fact that they rarely metastasize at distance,^9^ together with the low frequency of HLA class I loss of expression in CRC liver metastases, it is tempting to speculate that HLA class I is an important determinant of the metastatic behaviour of CRC. To investigate this further, we examined the metastatic patterns and HLA class I expression in the 4 metastatic tissues from MMR-d CRCs included in the NL cohort and in four additional cases retrieved from the tissue archive of the department of Pathology of the Leiden University Medical Center. From these, only two corresponded to liver metastasis while the remainder were diagnosed at various organs (Table 1). This is in clear contrast with the metastatic patterns from MMR-p CRCs that most often metastasize to the liver and lung. Further, 6/8 cases displayed loss of membranous HLA class I expression as determined by immunohistochemical detection (Table 1). While one liver metastasis retained membranous HLA class I expression, the other presented with cytoplasmic expression and was therefore considered defective in antigen presentation. It is unclear whether cytoplasmic expression might still allow the occasional presentation of HLA class I at the membrane, thereby allowing the colonisation of the liver by CRC cells. The establishment of liver metastasis by HLA class I-negative tumour cell clones may be thwarted by the abundancy of NK cells in this tissue, as these innate lymphocytes play a fundamental role in recognising and eliminating cells with a “missing-self” phenotype.^10^ To further illustrate the difficulty of obtaining metastatic samples from MMR-d CRCs, we assessed the MMR status through immunohistochemical detection of PMS2 and MSH6 in 110 lung metastasis from CRC and failed to identify any MMR-d case (data not shown).

**Table 1:** Anatomical location and HLA class I phenotype of MMR-d CRC metastasis

	Location	HCA2	HC10	HLA class I status
MDM1	Liver	++/++	++/++	Positive
MDM2	Distant lymph node	++/++	++/++	Positive
MDM3	Abdominal wall	−/+	++/++	Loss
MDM4	Adrenal gland	−/+	+/+	Loss
MDM5	Biceps muscle	−/+	++/++	Loss
MDM6	Peritoneum	−/+	C/++	Loss
MDM7	Peritoneum	C/++	++/++	Loss
MDM8	Liver	C/++	C/++	Loss

Scoring: Tumour/Internal positive control. HLA class I expression was detected with the HCA2 and HC10 monoclonal antibodies

*−* negative, *+* weak positive, *++* strongly positive, *C* cytoplasmic

## Discussion

We have confirmed and extended previous findings that support the high prevalence of defects in the antigen processing and presentation machinery in MMR-d CRCs, particularly through the loss of HLA class I expression. Given the heterogeneity in responses to checkpoint blockade in this patient group we urge researchers to investigate the relation between HLA class I phenotypes and response to immunotherapy in CRC. As demonstrated, the interpretation of HLA class I phenotypes by immunohistochemistry is not trivial and therefore should be performed by experienced researchers. Furthermore, when possible, HLA class I phenotypes should be supported by genetic data confirming mechanistic defects while RNA expression levels appear insufficient to determine the HLA class I status of tumours. Finally, we propose that HLA class I expression is an important determinant of metastatic behaviour in CRCs based on two observations: (1) total loss of HLA class I expression is rare in CRC liver metastasis and (2) distant metastasis are infrequent in MMR-d cancers, particularly to the liver, while most tumours have lost HLA class I expression. The applicability of checkpoint blockade in the context of local recurrence or in the neo-adjuvant setting should be considered so that an increased number of MMR-d CRC patients can benefit from these immunotherapies.

## Supplementary information

Detailed exome sequencing method

## **Data availability:**

Data is available upon request.
