# Supplementary material for: Revisiting immune escape in colorectal cancer in the era of immunotherapy
Source: Br J Cancer. 2019 Mar 13;120(8):815–8. doi: 10.1038/s41416-019-0421-x (PMC6474276; doi:10.1038/s41416-019-0421-x)
Supplement: Supplementary file 1 — Detailed exome sequencing method [file 41416_2019_421_MOESM1_ESM.docx]

Exome sequencing

Genomic DNA was isolated from 17 metastatic CRC tissues for which snap-frozen tumour and healthy tissues were available. After shearing, sequencing libraries were prepared with the NEBNext® Ultra™ II DNA Library Prep Kit for Illumina (New England Biolabs, Ipswich, MA, US) according to manufacturer’s instructions. Exomes were captured with the IDT xGEN Exome target kit (Integrated DNA Technologies, Leuven, Belgium), according to manufacturer’s instructions. Paired-end, 150bp sequencing was performed on a HiSeq4000 sequencer (Illumina, San Diego, CA, US) to obtain a dataset of 11 Gb (37 million PE- reads of Illumina-filtered sequence data) per sample. Reads were aligned by using the Burrows-Wheeler Aligner 3 (BWA-mem version 0.7.15)^1^ algorithm with default parameters. Duplicate reads were removed using Picard Tools (http://picard.sourceforge.net). Genome Analysis Toolkit 7 (GATK version 3.8; Broad Institute, Cambridge, MA, USA,) was used for base quality recalibration. Subsequently, SNV and InDel were called using a combination of three popular software tools, muTect^2^, varScan^3^, and Strelka.^4^ The resulting .vcf files were combined into a single file using GATK CombineVariants. Integrative Genomics Viewer (IGV, Broad Institute)^5^ was used for visually inspecting variants. Manual review of aligned reads was used to reduce the risk of false positives and incorrect calls.

1. Li H, Durbin R. Fast and accurate short read alignment with Burrows-Wheeler transform. *Bioinformatics*. 2009;**25**:1754-60.

2. Cibulskis K, Lawrence MS, Carter SL, Sivachenko A, Jaffe D, Sougnez C, et al. Sensitive detection of somatic point mutations in impure and heterogeneous cancer samples. *Nat Biotechnol*. 2013;**31**:213-9.

3. Koboldt DC, Zhang Q, Larson DE, Shen D, McLellan MD, Lin L, et al. VarScan 2: somatic mutation and copy number alteration discovery in cancer by exome sequencing. *Genome Res*. 2012;**22**:568-76.

4. Saunders CT, Wong WS, Swamy S, Becq J, Murray LJ, Cheetham RK. Strelka: accurate somatic small-variant calling from sequenced tumor-normal sample pairs. *Bioinformatics*. 2012;**28**:1811-7.

5. Thorvaldsdottir H, Robinson JT, Mesirov JP. Integrative Genomics Viewer (IGV): high-performance genomics data visualization and exploration. *Brief Bioinform*. 2013;**14**:178-92.
